# Transvenous extraction of a left bundle branch area pacing lead and an attempt to reimplant it: A case report

**DOI:** 10.1016/j.hrcr.2024.06.017

**Published:** 2024-06-25

**Authors:** Takehiro Nomura, Tsuyoshi Isawa, Kosuke Onodera, Shigeru Toyoda, Kennosuke Yamashita, Taku Honda

**Affiliations:** ∗ Department of Cardiovascular Medicine, Heart Rhythm Center, Sendai Kosei Hospital, Sendai, Miyagi, Japan; † Department of Cardiovascular Medicine, School of Medicine, Dokkyo Medical University, Shimotsuga-gun, Tochigi, Japan; ‡ Department of Cardiovascular Medicine, Sendai Kousei Hospital, Sendai, Miyagi, Japan

**Keywords:** Pacemaker, Conduction system pacing, Left bundle branch area pacing, Transvenous lead extraction, Pacemaker infection, Lumenless lead

**Key teaching points**

• The left bundle branch area pacing (LBBAP) lumenless lead was easily extracted with manual traction and counterclockwise rotation.
• However, the lead was covered with translucent fibrous tissue and had myocardium attached to the tip, suggesting the possibility of injury to the myocardium or left bundle branch (LBB).
• Even after initially removing the LBBAP lead, re-LBBAP could once again be achieved. However, the effect on LBB conduction and the feasibility of re-LBBAP after an LBBAP lead extraction require further investigation.

## Introduction

The feasibility of left bundle branch area pacing (LBBAP) has been demonstrated mainly by some single-center clinical studies.^1^^,^^2^ Several multicenter and observational studies have suggested that LBBAP improves clinical outcomes, as compared to biventricular pacing, in patients with cardiac resynchronization therapy indications.^3^ However, there are concerns regarding the feasibility of the transvenous extraction of LBBAP leads deeply screwed into the ventricular septum. To the best of our knowledge, there have been no reports of attempting LBBAP once again after extracting the lead for a pacemaker infection; however, there have been some case reports of successful LBBAP lead extractions.^4^, ^5^, ^6^, ^7^, ^8^

## Case report

**Figure 1:**
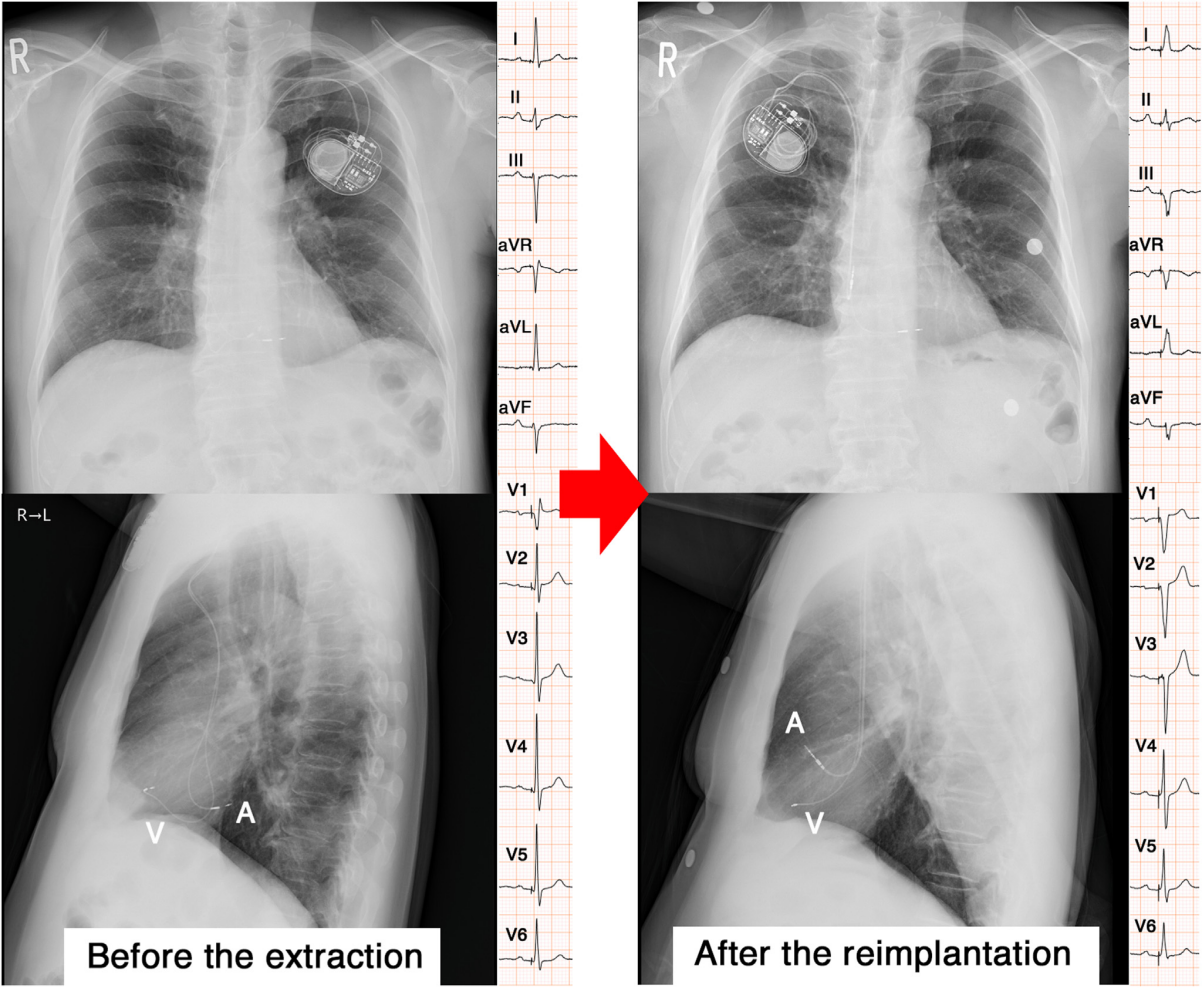
The electrocardiogram (ECG) before the lead extraction exhibited a right bundle branch delay pattern and the paced QRS duration and R-wave peak time (RWPT) in the V_6_ lead were 111 and 62 ms, respectively. The ECG after the reimplantation revealed a QS pattern in lead V_1_, and the QRS duration and RWPT in the V_6_ lead were 115 and 61 ms, respectively. The patient’s chest radiography images showed that the ventricular lead was slightly more advanced anteriorly than before the lead extraction.

**Figure 2:**
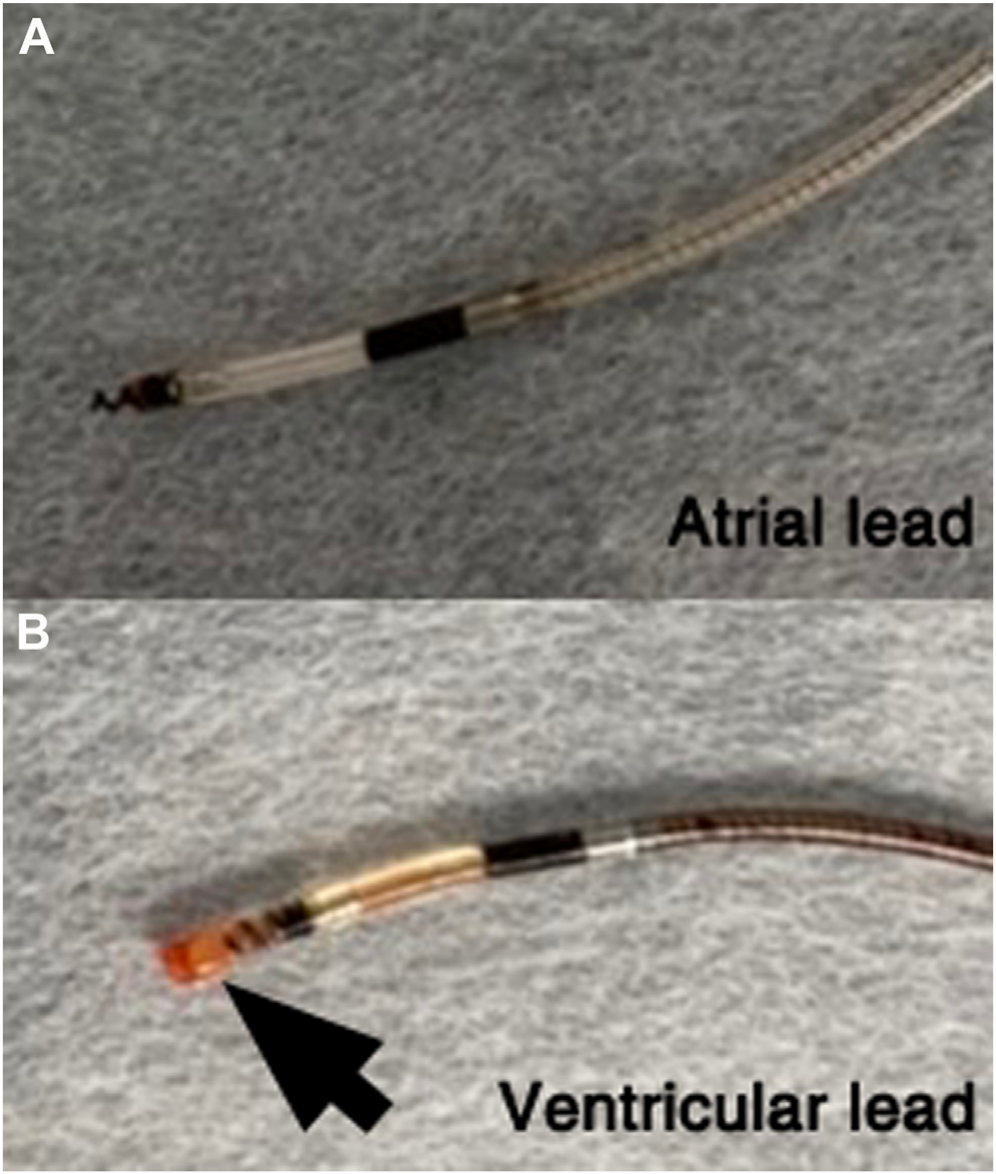
**A:** The extracted atrial lead tip was not broken and had no adherent material on the tip. **B:** On the other hand, the screw of the left bundle branch pacing lead was grossly covered with translucent fibrous tissue and had myocardium attached to the tip (*arrow*).

We present a case of a 72-year-old male patient with complete atrioventricular block and an old inferior myocardial infarction. He underwent LBBAP with a SelectSecure lead and C315HIS delivery catheter (Medtronic, Minneapolis, MN) at a local hospital. Diffuse redness and swelling over the pacemaker area were observed, and he was referred to our clinic with a diagnosis of a pacemaker pocket infection 6 months after the implantation. He had no fever but was diagnosed with a pocket infection because the pocket had perforated and discharged pus. His electrocardiogram (ECG) and chest radiography images are shown in Figure 1. The QRS duration was 111 ms and the R-wave peak time (RWPT) in the V_6_ lead was 62 ms with a voltage of 3.0 V and pulse width of 0.4 ms during bipolar pacing. The ventricular pacing rate was nearly 100% when the sensed and paced AV delay was 150 and 180 ms, respectively. Transthoracic echocardiography (TTE) revealed that his left ventricular ejection fraction (LVEF) was 52% and end-diastolic left ventricular diameter 43 mm. TTE also confirmed the atrial lead tip was located on the right atrial posterior wall. There were no moderate or further valvular abnormalities. His blood cultures were negative, but methicillin-sensitive *Staphylococcus aureus* was detected in the wound culture. Therefore, he underwent a transvenous lead extraction (TLE) procedure under general anesthesia. The intracardiac echocardiogram in the superior vena cava and right atrium revealed a lead-to-lead adhesion at the bifurcation of the innominate vein, but there was a little adhesion between the leads and the superior vena cava wall. Manual traction with counterclockwise rotation was successful and the lead became dislodged from the ventricular septum (Supplemental Video 1). However, the lead became stuck at the innominate vein bifurcation, probably owing to the lead-to-lead adhesion. It was decided to extract the atrial lead first, and manual traction with counterclockwise rotation was successful. After the atrial lead extraction, the ventricular lead became unstuck and was successfully extracted. The screw of the LBBAP lead was covered with translucent fibrous tissue and had myocardium attached to the tip (Figure 2). Temporary transvenous pacing was required after the ventricular lead extraction. No intrinsic QRSs occurred, even with an external pacing rate of 30 beats per minute during the period between the lead removal and the reimplantation. Postoperative TTE showed there were no complications including a ventricular septal perforation or pericardial effusion.

**Figure 3:**
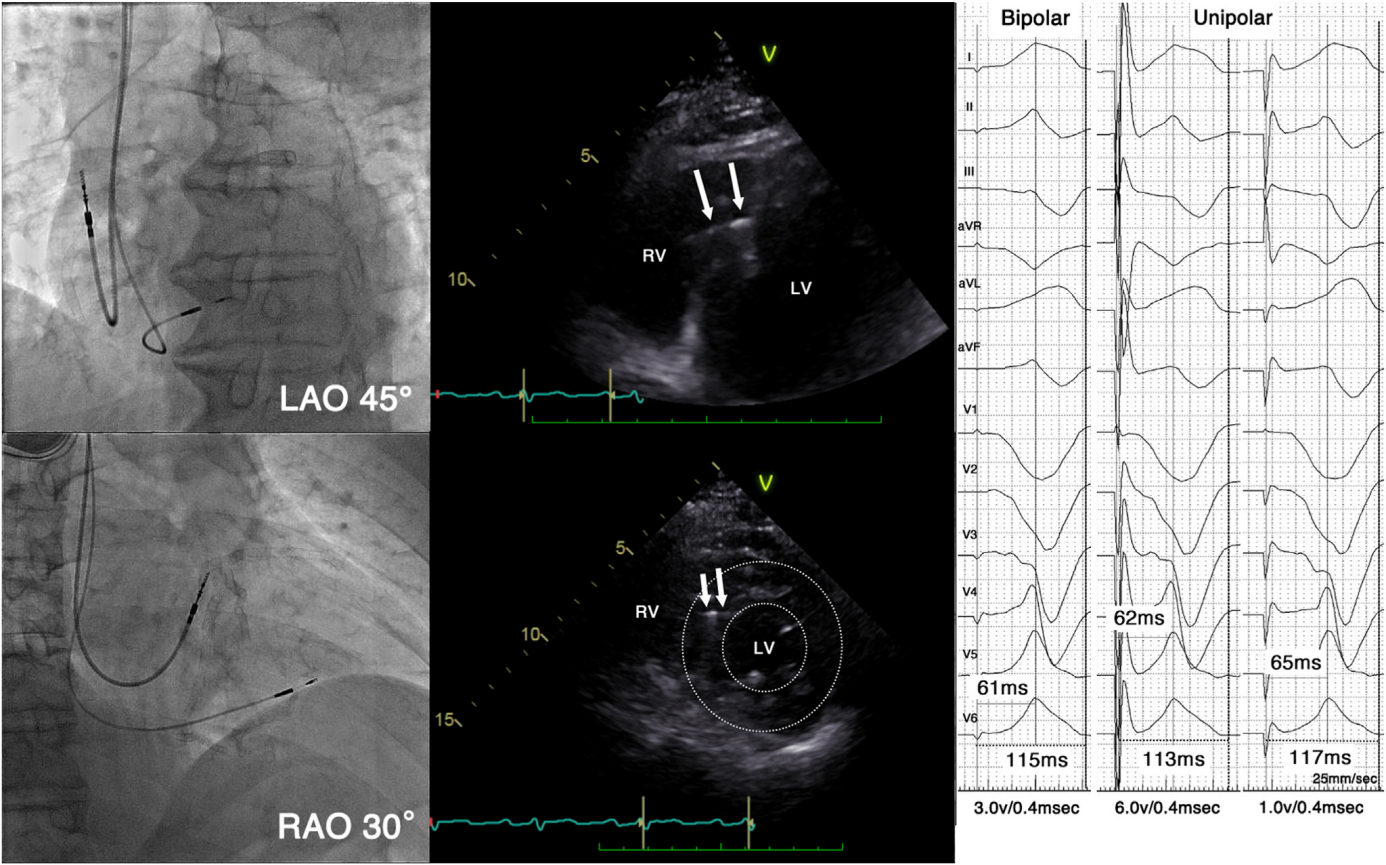
Fluoroscopy and transthoracic echocardiography images in right anterior oblique (RAO) and left anterior oblique (LAO) views showing the pacing lead location after the pacemaker reimplantation. The transthoracic echocardiographic images showed that the ventricular lead was not completely perpendicular to the septal myocardium but had advanced anteriorly and toward the apex (arrow). The R-wave peak time (RWPT) in lead V_6_ was 61 ms with a bipolar voltage of 3.0 V with a 0.4 ms pulse width, which was almost the same value as that before the lead extraction. The RWPT increased rather than decreased as the pacing stimulus amplitude decreased from 6 V to 1 V. LV = left ventricle; RV = right ventricle; V = volt.

The day after the TLE procedure, a new pacemaker was implanted on the right chest wall using a SelectSecure and C315HIS, as before the TLE. No paced QRS morphology with a “w” pattern with a notch in the V_1_ lead during unipolar tip pacing was identified at multiple sites of the right ventricular septum. We anatomically determined the ventricular lead position using the 9-partition method.^9^ The unipolar paced QRS complex had a QS pattern in the V_1_ lead when the lead was screwed about 10 mm into the septum in the left anterior oblique 45° view (Figure 1). The QRS duration and RWPT in the V_6_ lead were 115 and 61 ms, respectively, with a bipolar voltage of 3.0 V and 0.4 ms pulse width (Figure 3), which were nearly equal to those before the TLE. With a unipolar configuration, they were 115 ms and 62 ms with 6.0 V and a 0.4 ms pulse width and 117 ms and 65 ms with 1.0 V and a 0.4 ms pulse width (Figure 3). The QRS electrical axis was -30 and -24 degrees before the TLE and after the reimplantation, respectively. The bipolar pacing threshold with a 0.4 ms pulse width was 0.50 V. The lead was fixed at that position because shortening the procedure time was considered paramount to prevent a reinfection. The atrial lead was fixed in the right atrial appendage, and the pacing threshold with a 0.4 ms pulse width was 0.75 V. The procedural time was 75 minutes. The TTE images after the reimplantation did not show any evidence of a ventricular septal defect. His chest radiography images showed that the ventricular lead was slightly more advanced toward the anterior wall than before the lead extraction (Figure 1). The patient was discharged after 2 weeks of administering of cefazolin at a daily dose of 6 grams, as the infected wound had healed well.

## Discussion

There have been some case reports of successful LBBAP lead extractions.^4^, ^5^, ^6^, ^7^, ^8^ Most of the reports have described successful extractions through simple traction and counterclockwise rotation. Similarly, the lead tip was also easily dislodged with simple traction involving counterclockwise rotation in our present case. It has been reported that LBBAP leads are not firmly fixated to the myocardium, and the lead tip is only lightly fixated.^5^ It has been suggested that the SelectSecure lead can be more successfully extracted than conventional pacing leads.^10^ However, those case reports included patients in whom the pacing leads were surgically removed^5^ or mechanical-powered sheaths were used.^8^ In our present case, the screw of the LBBAP lead was also grossly covered with fibrous tissue and had myocardium attached to the tip. There has been a report of the tip of the SelectSecure lead being torn off and remaining adhered to the interventricular septum after the extraction.^6^ At least in some cases, the screw of the SelectSecure lead probably becomes tightly adhered to the ventricular septum during LBBAP. Therefore, the manual traction and counterclockwise rotation might be a procedure involving “twisting off” the lead and surrounding tissue rather than simply “unscrewing” it in patients with adhesions. The procedure might lead to left bundle branch injury and block, which could potentially impact the outcomes when again attempting conduction system pacing. We attempted a reimplantation of the LBBAP lead; however, his LVEF slightly reduced to 52%. That was because the ventricular pacing burden was 100% and LBBAP can significantly reduce the QRS duration and improve the systolic function in patients with a mildly reduced LVEF.

A case of a late dislodgement and successful reimplantation of an LBBAP lead 2 years after the initial implantation has been reported.^11^ In our case, a right bundle branch delay pattern was not present on his ECG after the reimplantation, which did not meet the conventional LBBAP criteria.^12^ However, the RWPT in lead V_6_ was 61 ms, which was almost the same value as that during LBBAP with a V_1_ qR pattern as before the lead extraction. In addition, the EHRA clinical consensus statement on conduction system pacing states that LBBAP can be confirmed with an RWPT in V_6_ of ≤75 ms,^13^ and the RWPT was 61–65 ms during unipolar stimulation in our case. The intrinsic left bundle branch (LBB) conduction was unclear owing to permanent complete atrioventricular block, and no other diagnostic findings such as an abrupt decrease in the RWPT were observed. However, we determined that LBBAP was achieved according to the EHRA consensus statement.^13^ It is interesting that the ECG exhibited nearly an equal RWPT and mean electrical axis, but a different QRS transitional zone was obtained in the same patient. Those findings may be explained by the capture of a septal fascicle or retrograde conduction from the left bundle to the right bundle branch.^14^^,^^15^

LBB injury may also affect the LBB conduction, but it is difficult to fully understand the etiology of the ECG findings, partly because the intrinsic LBB conduction both before and after the TLE was unclear. The present case suggested re-LBBAP could be achieved even after the LBBAP lead extraction procedure. However, the effect of the TLE on the LBB conduction and feasibility of re-LBBAP after an LBBAP lead extraction requires further investigation.

## Conclusion

The LBBAP lead was easily extracted with manual traction and counterclockwise rotation; however, the lead was covered with translucent fibrous tissue and had myocardium attached to the tip. Even after initially removing the LBBAP lead, LBBAP could once again be achieved.

## Disclosures

All the authors have no conflicts to disclose.

## Appendix supplementary data

Supplemental Video 1 Manual traction with counterclockwise rotation was successful and the lead became dislodged from the ventricular septum.
